# Advancing Brain Research through Surface-Enhanced Raman Spectroscopy (SERS): Current Applications and Future Prospects

**DOI:** 10.3390/bios14010033

**Published:** 2024-01-10

**Authors:** Suzan Elsheikh, Nathan P. Coles, Ojodomo J. Achadu, Panagiota S. Filippou, Ahmad A. Khundakar

**Affiliations:** 1 National Horizons Centre, Teesside University, 38 John Dixon Ln, Darlington DL1 1HG, UKn.coles@tees.ac.uk (N.P.C.); o.achadu@tees.ac.uk (O.J.A.); p.philippou@tees.ac.uk (P.S.F.); 2 School of Health and Life Science, Teesside University, Campus Heart, Southfield Rd, Middlesbrough TS1 3BX, UK; 3 Translational and Clinical Research Institute, Newcastle University, Newcastle upon Tyne NE1 7RU, UK

**Keywords:** SERS, brain, neuropathology, Alzheimer’s, brain cancer, biomarkers

## Abstract

Surface-enhanced Raman spectroscopy (SERS) has recently emerged as a potent analytical technique with significant potential in the field of brain research. This review explores the applications and innovations of SERS in understanding the pathophysiological basis and diagnosis of brain disorders. SERS holds significant advantages over conventional Raman spectroscopy, particularly in terms of sensitivity and stability. The integration of label-free SERS presents promising opportunities for the rapid, reliable, and non-invasive diagnosis of brain-associated diseases, particularly when combined with advanced computational methods such as machine learning. SERS has potential to deepen our understanding of brain diseases, enhancing diagnosis, monitoring, and therapeutic interventions. Such advancements could significantly enhance the accuracy of clinical diagnosis and further our understanding of brain-related processes and diseases. This review assesses the utility of SERS in diagnosing and understanding the pathophysiological basis of brain disorders such as Alzheimer’s and Parkinson’s diseases, stroke, and brain cancer. Recent technological advances in SERS instrumentation and techniques are discussed, including innovations in nanoparticle design, substrate materials, and imaging technologies. We also explore prospects and emerging trends, offering insights into new technologies, while also addressing various challenges and limitations associated with SERS in brain research.

## 1. Introduction

The global burden of brain and central nervous system (CNS) disorders is significant, comprising around 70% in low- and middle-income countries, and neurological conditions are the leading cause of disability-adjusted life years, accounting for about 9 million deaths per year [1]. This is exacerbated by a global demographic shift to an aging population, heightening the growth of age-associated neurological disorders such as Alzheimer’s disease (AD), stroke, and Parkinson’s disease (PD) [2]. Moreover, while comparatively rarer, brain cancers cause significant mortality and morbidity across all ages and remain the primary cause of cancer-related mortality in children diagnosed at 0–14 years old [3]. The need for novel, quick, and reliable diagnostic tools for the early detection of brain diseases has thus never been greater.

Raman spectroscopy (RS) is a label-free method that provides a molecular signature of any type of biological sample, including tissue, live or fixed cells and biofluids for disease diagnosis. RS allows a sample’s biochemical structure to be fingerprinted by analyzing the molecular bond vibrations of its biocomponents and has been employed to detect subtle biomolecular changes, enabling comparisons between a variety of tissues and biofluids [4,5,6,7]. RS has shown considerable potential in understanding the diagnosis, progression and treatment response for a variety of brain disorders, including dementia-causing illnesses [8,9,10,11], Huntington’s disease [12], and stroke [13]. RS has also been used in cancer diagnosis to capture subtle changes in biomolecular composition, such as in DNA or protein, allowing comparison and discrimination between cancerous and non-cancerous tissues and between stages [14,15,16]. Notably, the recent introduction of portable Raman devices, which provide spectral measurements within minutes, may offer potential for point-of-care diagnostic testing [17]. Nevertheless, a major limitation of portable RS, and RS in general, is its relatively low signal intensity and consequent lower sensitivity compared to other techniques (such as mass spectrometry), resulting from a low-power laser source [18]. This factor, along with a long acquisition period, the occasional appearance of fluorescence background from biological samples, and time-consuming big data processing, have so far hampered the widespread clinical use of RS. Recent progress in ‘post-processing’ developments, combining RS with machine learning and AI approaches, has been successfully implemented in disease diagnostics. Indeed, our group recently demonstrated accurate discrimination between malignant glioma grades (III and IV) in tissue, cell models, and serum samples using RS combined with machine learning [15], in line with earlier findings in cancer [19] and AD [20]. However, the use of surface-enhanced Raman spectroscopy (SERS) may overcome many of the challenges associated with conventional RS, offering higher sensitivity thorough scattering enhancement and fluorescence quenching, to enable high resolution and improve the accuracy of data acquisition [21,22]. SERS thus presents promising opportunities for a rapid, reliable, and non-invasive method in neuropathology.

In this review, we explore the practical applications and innovations of SERS in understanding the pathophysiological basis of neurological disorders, focusing on AD and PD, stroke, and brain cancer. We will also examine the use of SERS in intraoperative brain procedures, particularly in guiding brain tumor surgery, along with its potential in the detection of neurotransmitters. Such advancements have the potential to significantly enhance the accuracy of clinical diagnosis and further our understanding of brain-related processes and diseases.

## 2. Fundamentals of SERS

SERS is a powerful analytical technique that enhances the signal of Raman scattering, allowing sensitive and selective molecular analysis. Consequently, the Raman signal is significantly amplified when the sample is near to roughened gold or silver metal surfaces or nanoparticles (NPs). Two primary SERS methods may be employed in biomedical applications: label-free [23,24] or labeled, both of which possess inherent advantages and disadvantages. The label-free method involves extracting chemical bond vibration information from biomolecules through direct interaction with the substrate nanostructure, thereby revealing the biomolecular composition of samples. Though simple to conduct, this approach is sometimes susceptible to signal interference [25]. The labeled method, however, relies on the incorporation of Raman reporter molecules with robust and distinct Raman signals, serving as SERS tags. This offers advantages such as high accuracy and relative quantitation but is complicated in terms of operation [26]. Regardless of approach, the design of sensitive and rational plasmonic NPs for SERS is imperative in ensuring the effectiveness of the interaction between the sample and the substrate, facilitating reliable and accurate results in biomedical applications. SERS allows structural fingerprinting of low-concentration analytes through the plasmon-mediated amplification of electrical fields or chemical enhancement [27]. The SERS enhancement factor may approach approximately 10^10^–10^11^ for highly optimized surfaces resulting from chemical and electromagnetic enhancement mechanisms [28]. Electrochemical enhancement primarily stems from the localized surface plasmon resonance of metal NPs [29], which induce intense enhancement of the electromagnetic field near the metal surface when interacting with incident light [27]. When analyte molecules are located within the vicinity of these electromagnetic hotspots, Raman scattering signals are dramatically amplified at nanometer-scale regions near the metal surface, boosting Raman signals by several orders of magnitude. Chemical enhancement in SERS is contingent upon molecule–metal interactions, which facilitate charge transfer between the energy levels of the adsorbate molecules and the metal NPs. This interaction leads to the formation of an adsorbate–metal complex, characterized by a significantly larger Raman scattering cross-section compared to the free molecules [30]. These interactions contribute to further signal enhancement that can vary based on the chemical properties of the molecules involved [30].

**Figure 1:**
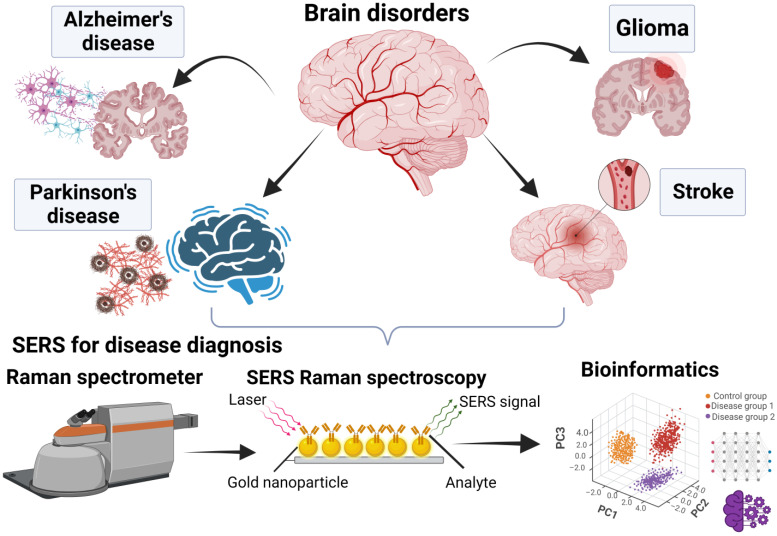
Surface-enhanced Raman spectroscopy (SERS) utility for a variety of brain disease diagnosis. SERS Raman spectroscopy enables the detection of biomolecular changes and combined machine learning techniques may improve the diagnostic performance. Created with BioRender.com; https://www.biorender.com, accessed on 16 November 2023.

The sensitivity of SERS allows analyte detection down to a single-molecule level. This has proved invaluable in applications where the analyte concentration is very low or where improving sensitivity is critical for accurate measurements, such as in the detection of biomarkers in complex biological samples [31]. This allows the identification and relative quantification of complex mixtures with high precision and could prove important in disease diagnostics, particularly when combined with bioinformatics and machine learning approaches (Figure 1).

## 3. Technological Advancements in SERS for Brain Research

The unique properties of SERS have heralded a new era in brain disease diagnostics. SERS-based approaches have been employed in both “bulky” measurements directly on the brain or surgical site to gain real-time information about tissues or conditions [32,33], along with indirect ex vivo analysis of biofluid samples, such as blood cerebrospinal fluid (CSF) or other bodily fluids, outside the living organism [13,34,35,36]. 

SERS analysis of liquid biopsies offers a less invasive diagnostic method compared to traditional tissue biopsies. [37,38]. SERS liquid biopsy allows dynamic monitoring of disease progression and treatment response in serum samples from cancer patients, for instance before and after tumor removal [39]. The SERS-based detection of circulating tumor cells (CTCs) [40] or cell-free DNA (cfDNA) [41] also provides a non-invasive means of early cancer detection. A key strength of liquid biopsies with SERS Raman technologies also lies in their multiplexed analysis capability [13,42,43,44,45], allowing the simultaneous detection of multiple biomarkers as a comprehensive molecular profile, within a single sample [44,46,47]. Nevertheless, SERS is subject to limitations according to sample handling conditions such as variability in sample preparation and handling procedures that may affect the performance and reproducibility of the results [48]. 

The primary challenge in the development of SERS technology for in vivo imaging is the acquisition of a signal from a SERS substrate located deep within the body. Initial research on in vivo SERS using rat or mouse models has employed mapping tools specifically designed to collect data from subcutaneously placed SERS probes [49,50,51,52,53,54]. However, a major drawback of SERS has been the lack of tissue penetration in the detection of brain pathologies, which often occur in deep-lying areas of the brain. NPs have been used in SERS to improve image contrast and target certain tissues and cells in vivo [50,51,53,54]. While SERS analysis is thorough and sensitive, its performance is dependent on the size, shape, and absorption properties of NPs. By incorporating diverse plasmonic materials into SERS spectroscopic methods, particular analytes of therapeutic relevance may be targeted. The shape of NPs may be optimized to provide maximal enhancement for various laser wavelength and Raman shift combinations. For example, nanorods with greater anisotropy outperform nanospheres and, in certain cases, nanostars in terms of SERS enhancement at longer wavelengths. According to Solís et al [55], nanorods, particularly at the frequently used laser wavelength of 785 nm, are an excellent option for developing extremely efficient SERS substrates.

**Figure 2:**
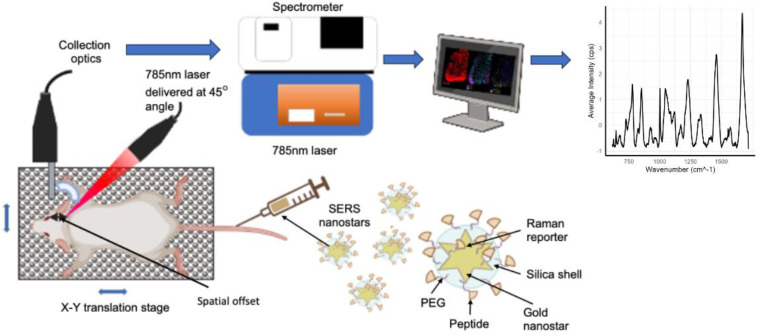
Concept and characterization for in vivo imaging of brain pathology using surface-enhanced Raman spectroscopy (SERS); for example, here, using surface-enhanced spatially offset resonance Raman spectroscopy (SESORRS) technology. This setup includes an XYZ translational stage, a laser positioned at a 45° angle relative to the collection optics, and a Raman spectrometer. Created with BioRender.com: https://www.biorender.com, accessed on 16 November 2023.

Innovative approaches have been employed to address the challenges of Raman imaging application to brain research. These cutting-edge technologies have included combining surface-enhanced resonance Raman spectroscopy (SERRS) and spatially offset Raman spectroscopy (SORS) to create surface-enhanced spatially offset resonance Raman spectroscopy (SESORRS), a method that allows imaging of the brain in vivo through the skull [53,56]. This entails the construction of specially designed SERS imaging equipment (summarized in Figure 2)**.** Gold nanostars functionalized with a resonant Raman reporter, forming SERRS nanotags, have been employed in a proof-of-concept work for SORS imaging of glioblastomas in transgenic mice models using a polytetrafluoroethylene (PTFE)-skull-tissue phantom [53]. The capacity to produce clear and distinct SERS spectra from deep-seated glioblastoma in mice in vivo through the skull was demonstrated using MRI and histology. SESORRS provided finer delineation of the tumor than standard Raman imaging [53]. This integrated technique is a huge step forward in overcoming the technological limits of optical-based brain imaging, particularly in terms of penetration depth. In light of such findings, Sharma et al [57] identified neurotransmitters in a brain tissue phantom via a cat skull, whereas Odion et al [58] recovered SERS spectra through a monkey skull using an inverse SORS technique. Using a portable SORS spectrometer, SESORRS has also been used to identify ex vivo multicellular tumor spheroids to depths of 15 mm of tissue [44,59].

Multimodal imaging approaches, although still uncommon, combine several imaging modalities presenting advantages over the traditional imaging techniques based alone on mass spectrometry (e.g. MALDI) or spectroscopy (e.g., SERS) alone. A recent multimodal imaging approach has integrated imaging modalities by employing a gold-coated nanostructured silicon substrate to couple surface-assisted laser desorption/ionization mass spectrometry (SALDI-MS) and SERS [60]. 

## 4. Applications of SERS in Brain Research

SERS offers potential in disease screening and diagnosis, with key studies emerging in the field of brain research. SERS has been investigated for use during brain surgery [61,62]. In glioma, which poses challenges in intraoperatively identifying its true margins due to its infiltrative nature, a SERRS probe has been developed [63], while a stimulated Raman scattering (SRS) microscopy method has accurately identified malignant tissue [64]. In the search for blood-based detection of AD biomarkers, a SERS-based sensor has developed for the relative quantitation of tau protein in the plasma of AD patients [65], while the combination of SERS with seed amplification assays (SAAs) offers an intriguing prospect in proteinopathies such as PD and AD [66]. A real-time assay for highly sensitive, label-free, multiplexed electrochemical, and SERS detection of stroke biomarkers has also been developed using a lateral flow device [13]. 

### 4.1. The Use of SERS in Glioma Research

Glioma accounts for more than 80% of all primary malignant brain tumors [67], and surgical removal is the mainstay of glioma treatment. However, due to the infiltrative nature of gliomas and the textural similarities between normal brain and malignant tissues, neurosurgeons face the challenge of maximizing the resection of the tumor while minimizing neurological deficits [68]. Though various approaches have been employed to identify brain tumor margins, few have truly defined the tumor’s infiltrative boundaries. RS has been used for discriminating between glioma grades when combined with machine and deep learning techniques [16,69,70]. Moreover, recent studies suggest SERS has the potential to precisely depict the actual tumor extent with high sensitivity, specificity, and spatial resolution, making it suitable for intraoperative image-guided resection [71].

SERS is emerging as a powerful tool in the realm of intraoperative brain mapping and real-time monitoring of gliomas. Various adjuncts have been proposed to aid neurosurgeons while operating on brain tumors to maximize the extent of resection in the surgical treatment of gliomas, including intraoperative fluorescence-guided microsurgery, intraoperative MRI (iMRI), intraoperative ultrasound (IOUS), intraoperative neuro-navigation, intraoperative frozen section, and intraoperative fluorescence-guided microsurgery [72]. While these methods have their merits, they also have limitations. Intraoperative MRI, for instance, requires specialized operating rooms and has time and environmental constraints. Conversely, intraoperative ultrasound is less expensive but has limitations in sensitivity for identifying residual tumor. RS, on the other hand, offers a non-invasive alternative approach with the ability to provide results in seconds, along with a highly sensitivity and specificity, and without the need for complex environmental requirements. 

So far, several animal studies have reported success in delineating glioma margins using a variety of SERS navigation systems [62,73,74,75]. Whole-brain tumor localization has been achieved in mouse models of glioma with a high degree of specificity and resolution using a combination of MRI, SERS and photoacoustic imaging, a technology that overcomes the depth and resolution limits of optical imaging [62,75]. Kircher et al. employed triple-modality imaging, combining MRI photoacoustic imaging, and Raman imaging [62]. NPs were intravenously injected into living mice with an orthotopic brain. This was facilitated through the disrupted blood-brain barrier, and NPs subsequently sequestered and retained by the tumor. This process resulted in accurate delineation of the margins of brain tumors, both preoperatively and intraoperatively [62]. 

More recently, a ratiometric pH-responsive SERS strategy has been developed for the rapid identification of glioma boundaries using pH-responsive SERS reporters [73,74] exploiting the pH gradient between glioma cells and extracellular fluid. The “Warburg effect” has also been used to characterize the metabolic anaerobic tendency of tumors, which results in significant lactic acid production [76]. SERS has been employed to identify tumor boundaries using a pH-sensitive SERS substrate, 4-mercaptopyridine (4-MPY), which reacts to pH changes. The lactic acid production in glioma cells lowers the local pH, impacting the 4-MPY SERS Raman peaks [74]. This approach has been tested using U87 cells in mice, yielding a surgical navigation system for tumor boundary identification. [73].

### 4.2. The Use of SERS in Alzheimer’s Disease Research

AD is the most prevalent type of dementia and is clinically characterized by significant amnestic cognitive decline, though can occasionally present as non-amnestic cognitive impairment. [77]. The AD brain exhibits microscopic features characterized by the abnormal accumulation of extracellular β-amyloid (Aβ) plaques and intraneuronal neurofibrillary tangles abnormally phosphorylated tau proteins (P-Tau) [78,79]. Using SERS to analyze AD biomarkers holds tremendous potential for accurate and early diagnosis. SERS-based biosensors have been developed, harnessing the optical properties of NPs to enhance detection performance [80]. Yu et al. have developed a sensitive SERS-based method to quantitatively detect serum biomarkers (such as Aβ1-42 and P-Tau-181) for early diagnosis of AD [81]. A SERS-based immunoassay successfully determined Aβ1-42 and P-Tau-181 in human serum, suggesting a promising tool for the early diagnosis of AD [81]. Since the detection AD biomarkers in blood has proven effective, the simultaneous analysis of multiple AD markers has been explored using SERS-based approaches. A lateral flow assay based on SERS nanotags (SERS-LFA) has been developed, allowing simultaneous quantification of multiple AD biomarkers, including Aβ42, Aβ40, tau protein, and neurofilament light chain, a marker of neuronal damage [82]. 

To monitor AD progression and rehabilitation treatments, an optimized protocol using SERS analysis of AD and control patient serum samples has been developed. The correlation of RS data with structural MRI demonstrated a direct link between Raman spectra and hippocampal degeneration, suggesting RS as a potential adjunct for monitoring AD diagnosis using scanning technologies [83]. The unique features of SERS, combined with SAAs, have also been shown to successfully improve amyloid β-oligomer detection and characterization of CSF of patients clinically diagnosed with AD. This approach has the potential to provide an early diagnostic test, complementing clinical evaluation and traditional laboratory tests [66]. Such early detection may improve the effectiveness of recently introduced drugs, such as aducanumab and lecanemab [84,85].

### 4.3. The Use of SERS in Parkinson’s Disease Research

PD is the most prevalent neurodegenerative movement disorder, characterized by the progressive development of bradykinesia, muscular rigidity, rest tremor, and postural instability [86]. These cardinal motor features stem from the gradual loss of dopaminergic neurons in the substantia nigra pars compacta. Along with other ‘synucleinopathies’ including dementia with Lewy bodies, multiple systems atrophy and pure autonomic failure, PD is defined by the presence of abnormal intracellular deposits termed ‘Lewy bodies’ and ‘Lewy neurites’ [87]. The main constituent of these pathological hallmarks is thought to be the unfolded protein α-synuclein, which is thought to aggregate from its native monomeric α-helical conformation, undergoing a profound conformational transition to a β-sheet-rich structure that form toxic oligomers and amyloid fibrils, accumulating as Lewy deposits [88,89,90]. 

**Figure 3:**
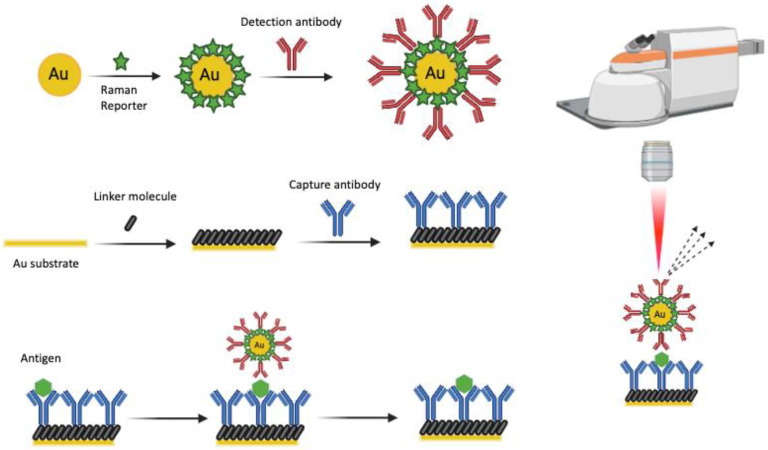
Labeled surface-enhanced Raman scattering (SERS)-based immunoassays for biomarker detection in biofluids for disease diagnostics. SERS-based immunoassay of protein (antigen) carried out by depositing biofluid samples on the Au substrate for immunocapture of the target antigen. SERS measurements performed in the antibody–antigen immunocomplexes. Created with BioRender.com: https://www.biorender.com, accessed on 16 November 2023.

The pathophysiology of PD and the development of effective treatments depend not only on the successful management of symptoms but also on targeting the underlying disease mechanisms and achieving early diagnosis before symptoms and clinical signs manifest. However, assessment results are often influenced by subjective and objective factors, which pose challenges to clinical diagnosis. Recent diagnostic advancements have harnessed the ‘prion-like’ properties of α-synuclein to develop SAAs, like the real-time quaking-induced conversion (RT-QuIC) assay, in various tissues and fluids, such as CSF [91] and, more recently, blood samples [92]. The combination of the RT-QuIC method with a targeted SERS-based immunoassay approach using antibodies directed toward specific proteo-forms of α-synuclein may thus offer an intriguing alternative approach in PD diagnostics (Figure 3). 

Multiplexed detection of biomarkers could hold huge potential in early diagnosis and personalized treatment of PD. Cao et al. fabricated a robust SERS-enabled lab-on-a-chip (LoC-SERS) platform for the simultaneous quantification of crucial PD-related proteins such as α-synuclein, P-Tau 181, osteopontin, and osteocalcin [35]. A multiplex amplification strategy has been developed to amplify the sensitivity of a lab-on-a-chip SERS system, allowing simultaneous and highly sensitive detection of miR-214 and miR-221, both potential biomarkers for the early-stage diagnosis of PD. [42]. Another study focused on 5-S-cysteinyl-dopamine (CDA), a crucial metabolite with high relevance for the early detection of PD [35]. This study involved assignment of SERS bands for CDA using silver NP substrates in aqueous media, with analysis supported by theoretical calculations and simulated Raman and SERS spectra [34].

### 4.4. The Use of SERS in Stroke Research

Globally, stroke continues to be the second most prevalent cause of mortality and the third most common cause of disability [93]. The rapid and reliable analysis of stroke is a primary goal for relevant therapeutic intervention. CT results may appear normal in the early stages of ischemic stroke or in patients with minor symptoms and MR imaging is often not feasible [94]. Although many blood biomarkers have been suggested for stroke diagnosis [94], the need for more sensitive and specific biomarkers remains a priority.

SERS detection of stroke biomarkers is considered a robust approach to overcoming these limitations. In a recent study by Sun et al., a real-time assay for highly sensitive, label-free, multiplexed electrochemical SERS identification of stroke biomarkers, specifically neuron-specific enolase (NSE) and S100-β protein, was developed using a lateral flow device [13]. Another study used a novel gold–silver alloy nanobox (AuAgNB)@SiO2-gold nanosphere nanoassembly based on a core–shell–satellite structure for the SERS detection of S100 calcium-binding protein B protein (S100B) [95]. Zhang et al. developed a novel lateral flow assay based on Raman encoded core–shell SERS nanotags for the rapid relative quantification of three cardiac biomarkers in the early diagnosis of acute myocardial infarction [45]. 

### 4.5. The Use of SERS in Neurotransmitter Detection

Neurotransmitters are endogenous signaling molecules secreted by neurons affecting a receptor on a target cell. Precise and proportional neurotransmitter release is vital for normal brain function and imbalances in neurotransmission has long been proposed to underlie many psychiatric and neurological conditions, including major depressive disorder [96], schizophrenia [97], epilepsy [98], PD [99] and AD [100]. Therefore, the monitoring of neurotransmitter concentrations could offer an exciting prospect in the diagnosis, prognosis, and treatment monitoring of brain disorders. 

Lussier et al (2017) introduced a dynamic SERS nanosensor (D-SERS), by modifying a patch clamp nanopipette with gold nano-raspberries [101]. The nanosensor can be precisely positioned within specific regions containing analytes under a microscope, enabling concurrent measurements of ATP, glutamate, acetylcholine, gamma-aminobutyric acid (GABA), and dopamine. The acquired SERS spectra of these neurotransmitters were subsequently subjected to barcode data processing techniques. This D-SERS nanosensor represents a versatile and reliable tool for investigating the secretion profiles of neurons. Dopamine measurement in human serum was achieved through SERS detection using a gold nanostructure fabricated on a silicon wafer to enhance plasmon resonance. To enable this detection, 4-mercaptophenylboronic acid (4-MPBA) was employed as a reporter molecule capable of forming covalent bonds with dopamine. The constrained Raman mode of dopamine-bound 4-MPBA exhibited a directly proportional variation to dopamine concentration, suggesting the sensor possesses high sensitivity and selectivity when applied to human serum for the purpose of dopamine detection [102]. 

Zheng et al (2023) recently developed a SERS-based method for neurotransmitters detection using gold-nanoislands, decorated tapered optical fibers with sub-10 nm gaps, enabling molecular fingerprint identification. The nonplanar repeated dewetting approach amplifies the high-density layer's broadband near-field amplification, allowing the detection of neurotransmitters without the use of exogenous reporters [103]. A SERS-active neural probe with gold nanoislands platform can detect neurotransmitters in the micromolar range, with a limit of detection of 10^−7^ M for rhodamine 6G and 10^−5^ M for serotonin and dopamine [104]. 

Quantitative SERS-based multiplexed detection of dopamine, serotonin and noradrenaline in human urine has been developed through chemometric analysis [43]. The consistent SERS signal intensities were a direct result of the precise sub-nanometer gaps between neighboring NPs. These findings indicate that this sensor has the potential for monoamine neurotransmitter detection in human urine at clinically relevant levels [43]. For serotonin, GABA, and glutamate, it was found that the lowest limits of detection were achieved using AgNPs SERS enhancing substrate at an excitation wavelength of 633 nm. In contrast, for indolic molecules like melatonin, dopamine, epinephrine and norepinephrine, the lowest limit of detection (LOD) was obtained with AuNPs at an excitation wavelength of 785 nm. This discrepancy is primarily attributed to the strong affinity of AuNPs to the indole ring [105]. 

## 5. Conclusions

In conclusion, SERS has considerable potential to shape brain research. Its potential to provide deeper insights into neurodegenerative disease and stroke pathology, diagnostics neurotransmitter measurement and brain tumor monitoring opens huge and exciting prospects. The emergence of portable SERS devices, machine and deep learning integration, and multimodal imaging promises more accessible and advanced methods for studying the brain. As these trends and prospects continue to evolve, they hold tremendous promise for advancing our understanding of brain function and pathology.

## Data Availability

Not applicable.
